# Acute Myeloid Leukemia: A Concise Review

**DOI:** 10.3390/jcm5030033

**Published:** 2016-03-05

**Authors:** Jennifer N. Saultz, Ramiro Garzon

**Affiliations:** 1 Medical Oncology/Hematology, Department of Internal Medicine, Starling-Loving Hall, Room M365, 320 W. 10th Ave., Columbus, OH 43210, USA; Jennifer.saultz@osumc.edu; 2 Division of Hematology, Department of Internal Medicine, 460 W 12th Ave, Columbus, OH 43210, USA

**Keywords:** AML, leukemia, review

## Abstract

Acute myeloid leukemia (AML) is a heterogeneous clonal disorder characterized by immature myeloid cell proliferation and bone marrow failure. Cytogenetics and mutation testing remain a critical prognostic tool for post induction treatment. Despite rapid advances in the field including new drug targets and increased understanding of the biology, AML treatment remains unchanged for the past three decades with the majority of patients eventually relapsing and dying of the disease. Allogenic transplant remains the best chance for cure for patients with intermediate or high risk disease. In this review, we discuss the landmark genetic studies that have improved outcome prediction and novel therapies.

## 1. Introduction

Acute myeloid leukemia (AML) is a heterogeneous disorder characterized by clonal expansion of myeloid progenitors (blasts) in the bone marrow and peripheral blood. Previously incurable, AML is now cured in approximately 35%–40% of patients younger than age 60 years old [1]. For those >60 years old, the prognosis is improving but remains grim. Recent studies have revealed that the disorder arises from a series of recurrent hematopoietic stem cell genetic alterations accumulated with age. Using deep sequencing techniques on primary and relapsed tumors, a phenomenon called clonal evolution has been characterized with both founding clones and novel subclones, impacting the therapeutic approach [2]. Despite an increased understanding of AML biology, our efforts to this point in changing treatment strategy have been disappointing. In this review, we discuss the current diagnostic and prognostic strategies, current treatment approaches and novel therapies critical to AML management.

## 2. Morphology

Morphologically, AML blasts vary in size from slightly larger than lymphocytes to the size of monocytes or larger. The nuclei are large in size, varied in shape and usually contain several nucleoli. AML blasts express antigens found also on healthy immature myeloid cells, including common differentiation (CD) markers CD13, CD33 and CD34 [3]. Other cells markers are expressed depending on the morphological subtype of AML and stage of differentiation block such as monocytic differentiation markers (CD4, CD14, CD11b), erythroid (CD36, CD71) and megakaryocytes markers (CD41a and CD61). On occasion, AML blasts also co-express antigens restricted to T or B cell lineages including Terminal deoxynucleotidyl transferase (TdT), Human leukocyte antigen-antigen D related (HLA-DR), CD7 and CD19. Rarely, the blasts can exhibit morphologic and immune-phenotypic features of both myeloid and lymphoid cells that make it difficult to classify them as either myeloid or lymphoid in origin. These cases are classified as mixed phenotypic leukemia and usually portend a worse overall survival [4]. Bone marrow aspirate and biopsy, including morphology, immune-phenotype, cytochemistry and genetics studies (conventional karyotype and molecular studies) remain essential for diagnosis, classification and risk stratification.

## 3. Classification

Over the years there have been several different classification systems for AML based on etiology, morphology, immune-phenotype and genetics. In the 1970s, AML was classified according to the French-American-British classification system using mainly morphology and immune-phenotype/cytochemical criteria to define eight major AML subtypes (FAB M0 to M7) [5]. The World Health Organization (WHO) classification of AML, replaced the old French-American-British classification system to become the essential modality for AML classification today. The WHO classification was updated in 2008 and identifies seven AML subtypes: (1) AML with recurrent genetic abnormalities (*RUNX1-RUNX1T1* t(8;21)(q22;q22), *CBFB-MYH11* Inv(16)(p13.1q22), t(16;16)(p13.1;q22), *PML-RARA* t(15,17)(q22;q12), *MLL* 11q23 abnormalities, *etc.*) and with gene mutations (Nucleophosmin 1 (NPM1) and CEBPA mutated gene); (2) AML with myelodysplasia-related changes; (3) Therapy related myeloid neoplasms; (4) AML not otherwise specified (NOS) (similar to FAB Classification M0–M7 with others such as acute megakaryoblastic leukemia, acute panmyelosis with myelofibrosis, and pure erythroleukemia); (5) Myeloid sarcoma; (6) Myeloid proliferations related to Down syndrome; and (7) Blastic plasmocytoid dendritic cell neoplasm [6]. Based on etiology alone, AML can also be subdivided into three distinct categories: (1) Secondary AML (s-AML) (associated with antecedent myelodysplastic syndrome (MDS) or other myeloid proliferative disorder (MPD)); (2) Therapy-related AML (t-AML) (associated with prior toxin/chemotherapy exposure) and (3) *De novo* AML [7].

## 4. Cytogenetics

Non-random chromosomal abnormalities (e.g., deletions, translocations) are identified in approximately 52% of all adult primary AML patients and have long been recognized as the genetic events that cause and promote this disease [8]. Certain cytogenetic abnormalities, including the t(8;21)(q22;q22), t(15;17)(q22;q12) and inv(16)(p13.1;q22) are associated with longer remission and survival, while alterations of chromosomes 5, 7, complex karyotype (described as >3 chromosomal abnormalities) and 11q23 are associated with poor response to therapy and shorter overall survival [1]. In contrast, about 40%–50% of all AML cases are cytogenetically normal (CN-AML) when assessed using conventional banding analysis [9]. Although, this group has an intermediate risk of relapse, a substantial heterogeneity is found in this population in terms of clinical outcome. Molecular screening of this AML category is critical for prognostic categorization and treatment strategy.

## 5. Molecular Abnormalities

During the last decade, several studies have shown that the presence or absence of specific gene mutations and/or changes in gene expression can further classify AML cases and have an effect on the patients’ prognosis [7,10,11]. As stated above, this is particularly relevant for patients with CN-AML. With the advent of next generation sequencing, the genetic landscape of CN-AML has been more defined with each case having an average of 13 mutations, eight of which are random “passenger” mutations and five of which are recurrent “driver” mutations [10]. Key molecular abnormalities have been identified and are now used to predict outcome and help guide treatment for AML patients. In the next sections we will describe the most relevant AML mutations discussed in relative order of frequency.

### 5.1. Nucleophosmin 1 (NPM1) Mutations

Nucleophosmin 1 (*NPM1*) mutations are the most frequent mutation in AML, occurring in 25%–30% of AML patients, with female predominance [12,13]. *NPM1* mutations result in the aberrant expression of the *NPM1* protein in the cytoplasm rather than the nucleus, stimulating myeloid proliferation and leukemia development [13,14,15]. Clinically, the mutation is associated with monocytic morphology and in the absence of FMS-like tyrosine kinase 3 or *FLT3-ITD*, predicts favorable overall survival (OS). The reason for improved survival remains unclear however it has been found that *NPM1* mutations have been associated with chemosensitivity to intensive chemotherapy in both young and old patients, which may account for improved outcome [16]. *NPM1* mutations are associated with other recurrent genetic abnormalities such as +8, *DNMT3A* mutations, *FLT3-ITD* (40% of the time), *FLT3-TKD* (10%–15%) and *IDH* mutations (25% of time) [11,17].

### 5.2. DNA Methyltansferase 3A (DNMT3A) Mutations

Mutations in the DNA methyltansferase 3A (*DNMT3A)* gene occurs in 18%–22% of all AML cases and in about 34% of CN-AML [18]. Missense mutations affecting arginine codon 882 (R882-DNMT3A) are more common than those affecting other codons (non-R882-DNMT3A) causing a defect in normal hematopoiesis and proper methylation [17]. Recently, DNMT3A mutations have been identified as pre-leukemic mutations, arising early in AML evolution and persisting in times of remission [19]. The prognostic significance of *DNMT3A* mutations is therefore thought to be adverse. Initial studies showed unfavorable impact on outcome in CN-AML [17]. However, these effects were age related. Younger patients with non-R882-DNMT3A mutations had shorter disease free survival (DFS) and overall survival (OS), whereas older patients with R882-DNMT3A mutations had shorter DFS and OS after adjustment for other clinical and molecular prognosticators [17]. A larger study involving more than 1700 AML cases found no significant impact of DNMT3A mutations on survival end points [20]. Recently, it was reported that patients with *DNMT3A*-mutated AML have an inferior survival when treated with standard-dose anthracycline induction therapy. Sehgal *et al.*, concluded that this group should be considered for high-dose induction therapy [21]. High-dose daunorubicin, as compared with standard-dose daunorubicin, improved the rate of survival among patients with *DNMT3A* or *NPM1* mutations or *MLL* translocations (*p* = 0.001) but not among patients with wild-type *DNMT3A*, *NPM1*, and *MLL* (*p* = 0.67) [22].

### 5.3. Fms-Like Tyrosine Kinase 3 (FLT3) Mutations

First described in 1991, FLT3 was found to be strongly expressed in hematopoietic stem cells with important roles in cell survival and proliferation [23,24]. Internal tandem duplications (ITD) in the juxta-membrane (JM) domain or mutations in the second tyrosine kinase domain (TKD) of the FLT3 gene have been found in 20% of all AML cases and 30% to 45% of CN-AML patients [1,25]. Both types of mutations constitutively activate FLT3 signaling, promoting blast proliferation [25,26]. Indeed patients with FLT3 mutations often present with extreme leukocytosis and characteristic prominent nuclear invagination often described as “cuplike” nucleus [25,27]. Furthermore, FLT3-ITD mutations have been associated with increased risk of relapse, while the prognostic relevance of FLT3-TKD mutations is controversial [28]. The degree to which FLT3-ITD is a biomarker associated with poor outcome is determined by the binding site and FLT3-ITD allelic burden [25,28,29]. Studies have shown that non-JM ITD are worse than JM domain ITD and higher mutant to wild-type allelic ratios were significantly associated with lower complete remission (CR) rates [28,29]. Currently, tyrosine kinase inhibitors (TKI) are being tested in FLT3 mutated AML patients. Unfortunately, when used alone, TKIs showed only a transient reduction of blasts, and even if initially effective, subsequent acquisition of secondary mutations induces resistance over time [30].

### 5.4. Isocitrate Dehydrogenase (IDH) Mutations

Mutations of the isocitrate dehydrogenase (*IDH*) 1 and 2 gene are gain-of-function mutations which cause loss of the physiologic enzyme function and create a novel ability of the enzymes to convert α-ketoglutarate into 2-hydroxyglutarate. *IDH* mutations are oncogenic. Specifically recurrent mutations affecting the highly conserved arginine (R) residue at codon 132 (R132) of *IDH1* and at codons *R140* and *R172* of *IDH2* have been identified in 15%–20% of all AML and 25% to 30% of patients with CN-AML [11,22,31]. They are found more frequently in older patients [32]. *IDH* mutations, in particular *IDH1*, are associated with lower DFS and OS in CN-AML cases with *NPM1* mutations and wild type *FLT3* [31,32]. Orally available, selective, potent inhibitors of mutated *IDH* are currently being tested in Phase I and II studies in AML with promising results [33].

### 5.5. Ten–Eleven Translocation 2 (TET2) Mutations

The ten–eleven translocation oncogene family member 2 *(TET2)* is found mutated in about 9%–23% of AML patients [34]. *TET1* is an enzyme involved in the conversion of 5-methylcytosine (5mC) to 5-hydroxymethylcytosine (5hmC) in DNA, which is a process thought to play an important role in DNA demethylation [34]. In general, *TET2* mutations are loss-of-function mutations. Overall, despite several studies their prognostic significance remains unclear. Metzeler *et al.*, reported *TET2* mutations as an adverse factor for CR and OS [35]. However Gaidzik *et al.*, did not show a prognostic effect with *TET2* mutations [36].

### 5.6. Runt-Related Transcription Factor (RUNX1) Mutations

Runt-related transcription factor (*RUNX1*) has been shown to be essential in normal hematopoiesis [37]. Also known as AML1 protein or core-binding factor subunit α-2 (*CBFA2*), *RUNX1* is located at chromosome 21 and is frequently translocated with the *ETO/MTG8/RUNX1T1* gene located on chromosome 8q22, creating a fusion protein *AML-ETO* or t(8;21)(q22;q22) AML [38]. In addition to chromosome translocations, *RUNX1* mutations are found in 5%–13% of AML and are commonly associated with trisomy 13, trisomy 21, absence of *NPM1* and older CN-AML [11]. In general, studies have shown *RUNX1* mutations are associated with resistance to standard induction therapy with inferior overall survival for both younger and older patients [39].

### 5.7. CCAAT Enhancer Binding Protein α (CEBPA) Mutations

The differentiation-inducing transcription factor CCAAT enhancer binding protein α (CBPA) mutations are found in 6%–10% of all AML and 15%–19% of CN-AML, commonly in association with del(9q) [1,40]. *CEBPA* is a critical transcription factor that controls gene expression during hematopoiesis [41]. In AML, *CEBPA* mutations commonly harbor two mutations or double mutations, which frequently involve both a combination of an N-terminal and a *bZIP* gene mutation. Importantly, only bi allelic mutation, not single, *CEBPA* mutations predicted a higher complete response (CR) and favorable OS, occurring in 4%–5% of AML [42]. AML with a single *CEBPA* mutation is associated with survival similar to that of AML with wild-type *CEBPA* [11,43].

### 5.8. Additional Sex Comb-Like 1 (ASXL1) Mutations

Additional sex comb-like 1 *(ASXL1)* mutations are loss-of-function mutations that occur in 5%–11% of AML cases [44]. The function of *ASXL1* protein is not fully understood, but it is suggested that it may be involved in epigenetic regulation (DNA and/or histone modifications) [36]. *ASXL1* mutations are five times more common in older (≥60 years) patients (16.2%) than those younger than 60 years (3.2%; *p* < 0.001) [44]. Among older patients, *ASXL1* mutations are associated with t(8;21), wild-type *NPM1*, absence of *FLT3-ITD*, mutated *CEBPA*, and overall inferior complete remission and overall survival [45,46].

### 5.9. Mixed Lineage Leukemia (MLL) Mutations

The mixed lineage leukemia (*MLL*) gene at chromosome 11q23 encodes for a protein that has histone methyltransferase activity that coordinates chromatin modification as part of a regulatory complex [47]. Translocations affecting the *MLL* gene lead to aggressive acute lymphoblastic and myeloid leukemia with poor prognosis that is characterized by *HOX* gene overexpression [37]. In addition to translocations, partial in tandem duplications (PTD) of the *MLL* gene (*MLL-*PTD) have been demonstrated most often in adult *de novo* CN-AML and in trisomy 11 AML cases [48,49]. In adult CN-AML, the frequency of MLL rearrangement is 11% with the presence of the *MLL-*PTD associated with a worse prognosis (*i.e.*, shorter duration of remission) when compared with CN-AML without the *MLL-*PTD [50].

### 5.10. Tumor Protein p53 (TP53) Mutations

The tumor suppressor gene *TP53* is found in 8%–14% of AML cases. These mutations and deletions are primarily associated in AML with complex karyotype (69%) and are rare in patient without chromosomal deletions. In general, *TP53* mutations confer a very adverse prognosis with documented chemoresistance [51].

### 5.11. c-KIT Mutations

The KIT tyrosine kinase receptor is a 145 kDa transmembrane protein critical to normal hematopoiesis [52]. This mutation is rare in AML (<5%) however present approximately 22%–29% of the time in *CBF* mutations (*i.e.*, AML harboring t(8;21)(q22;q22) or inv(16)(p13.1q22) or corresponding respective fusion genes *RUNX1/RUNX1T1* and *CBFB/MYH11).* *KIT* mutations have been shown to confer higher relapse risk and lower OS. The *KIT* mutation in the codon *D816* in particular has been associated with unfavorable DFS and OS, particularly in t(8;21)(q22;q22) patients [53]. Prospective studies later confirmed that patients with *CBF* AML harboring *KIT* mutations have shorter OS than patients with wild type *KIT* for t(8;21)(q22;q22) but not for patients with inv(16)(p13.1q22) [54]. Remarkably *KIT* could be targeted pharmacologically by using tyrosine kinase inhibitors, such as dasatinb [52]. Preliminary results were presented recently at the American Society of Hematology Annual Meeting from a phase II trial that combined the *KIT* inhibitor, dasatinib with standard chemotherapy for newly diagnosed patients with *CBF* AML. After a median follow-up of 21 months, patients with *KIT* mutations who received dasatinib with standard chemotherapy showed similar outcomes to that of wild type *KIT* patients [55]. Unfortunately, no survival benefit was found with maintenance dasatinib in a phase II study completed by Boissel *et al.*, Interestingly, at relapse there was disappearance of the KIT subclone which is hypothesized to be dasatinib driven [56]. More studies are needed to evaluate the long term outcomes of *KIT* inhibitors in *CBF* AML.

### 5.12. Spilicing Factor Gene Mutations and Mutations in Cohesion Complex Members

Often considered founding mutations, spilicing factor gene mutations have been found to be associated with pre-leukemic conditions such as MDS. The most common genes reported include *SF3B1*, *U2AF1*, *SRSF2* and *ZRSR2* [7]. In newly diagnosed AML patients, splicesome mutations including *SRSF2*, *F3B1*, *U2AF1*, or *ZRSR2* are now considered pathognomonic of secondary AML developing from precedent MDS [57]. Somatic cohesion complex mutations were identified in roughly 20% of patients with high-risk MDS and secondary AML. Relevant mutations include *STAG2*, *TAD21* and *SMC3* which are important in regulating gene expression and DNA-loop formation. Mutations in cohesion complex members are associated with poor overall survival [58].

## 6. Prognosis/Risk Stratification

Age and performance status in addition to chromosomal and molecular aberrations remain the most important tools for outcome prediction in AML. In 2010, the European LeukemiaNet (ELN) classification scheme was created in an effort to standardize risk stratification in adult AML patients by incorporating cytogenetic and known molecular abnormalities [59]. Patients are classified into one of four risk groups: favorable, intermediate 1, intermediate 2 and adverse (Table 1). Favorable prognosis is associated with acute promyelocytic leukemia (APL) t(15;17)(q22;q12), balanced abnormalities of t(8;21)(q22;q22), inv(16)(p13.1q22), t(16;16)(p13.1;q22), mutated *NPM1* without *FLT3-*ITD and biallelic mutated *CEBPA*. Intermediate 1 includes mutated *NPM1* with *FLT3-*ITD, wild-type *NPM1* with or without *FLT3-*ITD. The intermediate -2 category includes t(9;11), *MLLT3-MLL* and cytogenetic abnormality neither favorable nor adverse. Complex karyotype, inv(3)(q21q26)/t(3;3)(q21;q26), *RPN1-EVI1*, *DEK-NUP214* t(6,9)(p23;q34), t(6;11), −5 or del(5q), −7 or abnormal (17p) and monosomal karyotype are associated with poor prognosis [59,60]. Patients with monosomal karyotype (defined as having two of more distinct monosomies or one monosomy and another structural abnormality) have a very poor prognosis (less than 4% survival at four years) [61]. Studies have shown that age >60 is an independent predictor of poor outcomes regardless of the ELN classification [60].

APL is risk stratified according to the risk of relapse based on initial white blood count (WBC) and platelet count at diagnosis. The following patient categories are: (1) low-risk: presenting WBC count below or equal to 10 × 10^9^/L and platelet count above 40 × 10^9^/L; (2) intermediate-risk: presenting WBC and platelet counts below or equal to 10 × 10^9^ and 40 × 10^9^/L, respectively; and (3) high-risk group: presenting WBC greater than 10 × 10^9^/L. Treatment strategy varies depending on risk stratification at diagnosis however, the inclusion of arsenic trioxide (ATO) in frontline therapy seems to benefit all-risk category APL patients [62].

## 7. Therapeutics

### 7.1. Induction Therapy

Since 1970, the backbone of intensive induction chemotherapy remains unchanged. For young adults (age < 60 years) and fit elderly patients (especially those harboring *NPM1* mutations and CBF leukemia) the intensive anthracycline and cytarabine regimen, “7 + 3”, induction therapy is the standard of care. The typical dose and schedule includes either daunorubicin (60 or 90 mg/m^2^ on days 1, 2 and 3) or idarubicin (10–12 mg/m^2^ on days 1, 2 and 3) given with seven days of continuous cytarabine infusion (100 mg/m^2^/daily for one week (days 1 through 7). The goal of induction chemotherapy is to achieve morphologic complete remission (CR), which is defined as: (1) <5% blasts in bone marrow aspirate sample with marrow spicules and with a count of ≥200 nucleated cells (no blasts with Auer rods or persistence of extramedullary disease); (2) absolute neutrophil count (ANC) >1000/µL, and (3) platelets ≥ 100,000/µL [63]. Young, *de novo*, AML patients achieve CR in 65%–73% using standard induction with “7 + 3” while only 38%–62% of patients over 60 years of age with AML achieve CR [64,65,66]. Several trials have now shown that higher dose of anthracycline (90 *versus* 45 mg/m^2^) in both younger and older fit adults (from 60 to 65) results in higher CR rates and increases the duration of OS [65,66]. Concerns about toxicity of high-dose daunorubicin and the wide use of the 60-mg/m^2^ dose as a newer “standard,” led the United Kingdom (UK) National Cancer Research Council (NCRC) to conduct a prospective randomized trial with the goal to compare daunorubicin at 60 *vs.* 90 mg/m^2^ in the induction of 1206 AML patients [67]. In this study there was no benefit of using higher dosing (90 mg/m^2^) over 60 mg/m^2^ across all subgroups [67]. However there are some caveats to consider in this trial. In particular, the cumulative dose of anthracyclines in the low dose arm (60 mg/m^2^) was equivalent in the United Kingdom National Cancer Research Institute (UK NCRC) trial to the higher dose (90 mg/m^2^) of the other clinical trials due to multiple courses of anthracycline. In addition the UK NCRC trial has a shorter follow up [68]. Thus, it is clear that 45 mg/m^2^ of daunorubicin seems insufficient and 60 mg/m^2^ is not inferior to 90 mg/m^2^ with less associated toxicity. Patients found to have a FLT3 mutation should be treated with a FLT3 inhibitor (discussed in more detail below), such as midostaurin, added to standard induction therapy [69].

Characterizing fitness in the adult population is important when deciding treatment strategy. In particular, appropriate therapy in the elderly AML patient should be determined based on “patient-specific fitness” using geriatric assessments to determine fitness, vulnerable and frail status regardless of age [70]. In older adults, deemed not fit for intensive induction therapy especially harboring complex karyotype without *NPM1* mutations, the use of hypomethylating agents including decitabine and azacitidine has shown to be beneficial [70,71,72]. Both agents, commonly used to treat myelodisplasia, have activity in AML as initial induction therapy and in the relapsed setting. Several phase II and III studies using azacitadine and decitabine have been conducted [71,72,73]. A study of 82 patients with AML, median age of 72 years, who received azacitidine as part of a compassionate use program showed CR/incomplete CR in 11 of the 35 untreated patients (31%). The median overall response duration was 13 months with the one-year and two-years overall survival rates of 58% and 24%, respectively [73]. Blum *et al.* showed an even higher complete remission rate of 47% and overall response rate of 64% with 10 days of low-dose decitabine at 20 mg/m^2^ intravenous over 1 h [72]. This treatment was well tolerated with CR achieved in 52% of subjects presenting with CN-AML and in 50% of those with complex karyotypes [72]. Older patients receiving induction decitabine usually require a median of two to four cycles of therapy to have an optimal response.

Patients with suspected acute promyelocytic leukemia (APL) should be treated with all-trans retinoic acid (ATRA) even before the diagnosis is confirmed. Early use of ATRA decreases the risk of APL induced coagulopathy, development of disseminated intravascular coagulation (DIC) and mortality. For patients with low-to-intermediate-risk APL (WBC ≤ 10 × 10^9^/L) outcomes are excellent with the use of ATRA with arsenic (ATO) [74]. In this non-inferiority study, the ATO-ATRA combination showed CR rates in all 77 patients (100%) and in 75 of 79 patients (95%) in the ATRA-idarubicin group. The two-year event-free survival and OS rates were significantly improved (97% and 99%) in the ATO-ATRA arm than for those in the ATRA–chemotherapy arm (86% and 91%) [74]. For high-risk patients (WBC > 10 × 10^9^/L), chemotherapy with idarubicin should be initiated once the diagnosis is confirmed in addition to ATO-ATRA for rapid control of leukocytosis. During induction treatment it is recommended that WBC, fibrinogen level, prothrombin time and partial thromboplastin time be monitored at least twice daily with aggressive transfusion support (platelet count ≥ 30 × 10^9^/L and fibrinogen level ≥ 1.5 g/L). Prophylactic steroids are also recommended, in particular when using ATRA/ATO combination for induction in patients with high WBC count to prevent differentiation syndrome [74,75].

### 7.2. Consolidation Strategies

Consolidation or post-induction therapy is given to prevent relapse and eradicate minimal residual leukemia (MRD) in the bone marrow after induction as a bridge to transplant or to achieve cure. Assessment of minimal residual disease using real-time PCR or Next Generation Sequencing (NGS) is increasingly being used to help track treatment response and has been shown to be superior than morphology alone in predicting impending relapse [76,77]. Despite this powerful information, the heterogentiy of AML in general has made following mutational clones difficult to determine absolute risk of leukemia development as some clones can persist in patient in long-term remission following treatment, such as *DNMT3A* [19]. In general, there are two main strategies for consolidation; chemotherapy (including targeted agents) and hematopoietic stem cell transplantation [64]. Both strategies could be used alone or most commonly in combination depending on the type of leukemia, the fitness of the patient and the availability of a stem cell donor. Post induction chemotherapy using intermediate-dose cytarabine 1.5 g/m^2^ twice daily on days 1, 3 and 5 given in three to four cycles is an effective and established regimen to prolong remission and improve survival in favorable risk young adults (<60 year of age) [8]. These patients are usually treated with chemotherapy alone and transplantation is reserved only at relapse [64]. In 2013, Burnett *et al.* challenged this dose schedule for adults <60 year old and showed that higher dose (3 g/m^2^) as compared to lower dose (1.5 g/m^2^) cytarabine for three courses led to identical outcomes [78]. Thus, low dose cytarabine at 1.5 g/m^2^ became the standard of care. High-dose cytarabine is still used for patients with CBF AML [e.g., t(8:21); or inv(16)] and NPM1 mutated AML [8,78]. In elderly patients (>60 year of age) there was no benefit with high dose cytarabine with increased and sometimes irreversible neurotoxicity noted [79], therefore 500–1000 mg/m^2^ is standardly used [1].

For other prognostic groups, in particular fit patients with intermediate risk or high risk disease after achieving CR, allogeneic hematopoietic stem cell transplantation remains the most effective long term therapy for AML with cure in 50% to 60% of patients in first CR [80,81]. Despite this, several patients never become eligible for transplant given co-morbidities, failure to achieve CR or lack of suitable donor [80]. While waiting for transplant it is standard practice to give post induction chemotherapy to maintain CR and keep the leukemia burden low. Decisions regarding consolidation rather than moving straight to transplant should be individualized as consolidation therapy poses risk of morbidity and mortality, which may hinder eventual curative transplant. Recent evidence unanimously confirms that age should no longer be used as the sole criteria for transplant eligibility [80,82]. Rather eligibility should be decided upon based on pre-transplant performance status, co-morbidities and current remission. The most widely recognized and validated tool for assessing comorbidity includes the Hematopoietic Cell Transplantation Comorbidity Index (HCT-CI) [82]. The higher the comorbidity index score, the worse the clinical outcome. Improvements in supportive care, increased donor options (haplo-identical donors and cord grafts) and reduced intensity preparation regimens for HCT have increased the success of transplant in all age groups. It is for this reason that we advocate for early patient discussion, risk assessment and tissue typing at diagnosis. Conditioning regimen should be decided based on patient fitness, transplant options and disease characteristics. Although risk of relapse is higher, long term outcomes of reduced-intensity allogeneic hematopoietic stem cell transplant in patients who were ineligible for myeloablative transplant are promising [81]. The results of a prospective multicenter phase II trial conduced by the Alliance for Clinical Trials in Oncology (formerly Cancer and Leukemia Group B) and the Blood and Marrow Transplant Clinical trial Network showed reduced intensity conditioning-based hematopoietic stem cell transplant (HSCT) to be an effective strategy for suitable older patients with an available matched donor with a disease-free survival and OS at two years after transplant of 42% and 48%, respectively [83]. Reduced intensity transplants are therefore becoming more common and clinically accepted.

### 7.3. Relapsed Disease

Of the patients who relapse, only a small fraction achieve successful second remission using salvage chemotherapy followed by allogeneic stem cell transplant with curative intent [64]. Studies examining clonal evolution of relapse show that relapse can occur from expansion of major or minor clones present at diagnosis or through newly acquired mutations over time [2]. Therefore, clinical trial is the preferred treatment approach especially in light of novel targeted therapies. Early relapse (occurring within the first six months after CR1) portends a poor overall survival. Salvage regimes include intermediate dose cytarabine (500–1500 mg/m^2^ intravenously every 12 h on days 1–3); MEC (Mitoxantrone 8 mg/m^2^ on days 1–5, Etoposide 100 mg/m^2^ on days 1–5, and Cytarabine 100 mg/m^2^ on days 1–5) or lastly, FLAG-IDA (Fludarabine 30 mg/m^2^, intravenously on days 1–5 (20 mg/m^2^ in patient >60 years old), Cytarabine 1500 mg/m^2^ (500–1000 mg/m^2^ in patients >60 year) intravenously, 4 h after fludarabine infusion, on days 1–5; Idarubicin 8 mg/m^2^, intravenously, on days 3–5; Granulocyte colony-stimulating factor 5 μg/kg, subcutaneously, from day 6 to white-cell count >1 g/L (FLAG-IDA) [1]. The likelihood of achieving a second CR is best in patients with a long first remission, younger age and in those with favorable cytogenetics [84]. In cases of APL, re-induction with ATO with or without ATRA remains the standard. CR rates with single agent ATO are good at roughly 85% [85].

## 8. Novel Targets

### 8.1. Fms-Like Tyrosine Kinase 3 (FLT3) Inhibitors

Several *FLT3* small molecule inhibitors have been developed with mixed results. First generation drugs include multi-kinase inhibitors such as midostaurin, lestaurtinib, tandutinib sunitinib and sorafenib. When used as single agents they have limited anti-leukemia activity mostly showing only transient reduction of blood and bone marrow blasts and increased toxicity [86]. In a randomized trial of 224 patients with *FLT3* mutated AML in first relapse lestaurtinib did not increase the response rate or prolong survival [87]. Single agent use with midostantrum, tandutinib and KW2449 in phase I/II trials were also not clinically effective [88,89,90]. Combination therapy using *FLT3* inhibitors with chemotherapy have also been conducted. Serve *et al.* reported a randomized trial of 201 newly diagnosed older AML patients, using the addition of sorafenib to induction and consolidation therapy. Unfortunately, sorafenib did not improve outcomes and patients did worse in the sorafenib arm due to higher treatment-related mortality and lower CR rates [91]. A recent phase II study of sorafenib in combination with 5-azacitadine in relapsed/refractory *FLT3-*ITD mutant AML demonstrated a response rate of 46%, mostly consisting of CR or CR with incomplete count recovery [92]. Sunitinib added to induction and consolidation chemotherapy in older patients with AML and *FLT3* activating mutations showed some effectiveness with CR rates 53% (8/15) and 71% (5/7) for patients with *FLT3-*ITD and *FLT3-*TKD mutations, respectively. The 13 patients who achieved CR went on to be consolidated with high dose cytarabine and 7/13 received sunitinib maintenance. The median overall survival in this study was 18.8 months [93]. The largest randomized, phase III clinical trial in FLT3-mutated AML conduced to date was recently presented at the 2015 American Society of Hematology (ASH) Plenary session showing the benefit of midostaurin added to induction chemotherapy (RATIFY trial) in which patients receiving midostaurin had significantly longer median OS than those receiving placebo: 74.7 *versus* 25.6 months (*p* = 0.0076) [94]. Second generation agents, promising to have better potency and less side effects include quizartinib and crenolanib are still undergoing clinical investigation. One trial, using quizartinib (AC220), did show better blast count clearance however also noted the development of secondary resistance. Drug resistance has since become the major challenge in treating patients with a single *FLT3* inhibitor. The point mutations identified which lead to resistance include *N676*, *F691*, and *D835* within the kinase domain of *FLT3-*ITD [95]. The novel *FLT3* inhibitors, G-749 and ASP2215 (active against both FLT3 ITD and D835 mutations), have recently been shown to provide sustained inhibition of *FLT3* phosphorylation and increased ability to overcome drug resistance in pre-clinical trials but further studies are needed to determine if it will have clinical efficacy [96,97].

### 8.2. Isocitrate Dehydrogenase (IDH) Inhibitors

The *IDH1* inhibitor AG-120 and the *IDH2* inhibitor AG-221 have demonstrated promising response rates in patients with AML in two separate phase I clinical trials [98,99]. Preliminary results were recently presented for both trials. The objective response rate (ORR) with AG-221 was 40% and 31% with AG-120 in relapsed/refractory AML patients. More interestingly the duration of the responses for AG-221 and AG-120 were more than 15 and 11 months at the analysis, and remained ongoing. Overall, 76% of responses lasted longer than six months. Based on these data, the Food and Drug Administration (FDA) have granted the medication an orphan drug designation for patients with AML.

### 8.3. Nuclear Exporter Inhibitors

The anti-leukemic efficacy of reversible inhibitors of the major nuclear export receptor, chromosome region maintenance 1 (*CRM1*, also termed *XPO1*) has brought much excitement. *CRM1* is a major nuclear exporter protein which mediates the export and inactivation of several tumor suppressors such as p53, p73, FOXO1, RB1 and p21 (CDKN1A) among others [100]. *CRM1* has been shown to be upregulated in a range of solid tumors and hematological malignancies, including AML [101,102]. Preclinical studies indicate that treatment of AML cell lines, patient samples and AML xenografts with novel *CRM1* inhibitors (Selinexor) induces strong anti-leukemic effects [103,104]. Based on these studies, Phase I/II clinical trials are currently ongoing to assess the safety, tolerability and activity of selinexor in AML patients.

### 8.4. Immune Therapies

Novel antibody therapies are revolutionary in the treatment leukemia and currently under development in AML. Monoclonal antibodies being explored include CD33 (Gemtuzumab ozogamicin) [105] and bispecific antibodies such as AMG 330 (anti-CD33 and CD3) [106]. Chimeric antigen receptor (CAR)-transduced T cells (CARTs) are T cells engineered to express a specific antigen receptor target designed against a specific cell-surface antigen. CD123 has been found to be expressed on the majority of AML blasts but also normal hematopoietic cells. Preclinical data shows that targeting CD123 via CARTs results in rejection of human AML and myeloablation in the mouse models [107].

## 9. Conclusions

AML is complex disease with a diverse genetic landscape. The field is rapidly expanding with increased understanding of the biology as well as potential new drug targets. Despite our best efforts at targeted therapy, it has become apparent that single drug options may be less likely to succeed over multiple drug targets. Relapse disease remains the highest cause of mortality after HCT. Immunotherapy is also an exciting new therapeutic approach which may offer long term cures for relapsed patients. We remain hopeful that the therapeutic options will continue to improve, with less toxicity and improved efficacy.

## Figures and Tables

**Table 1:** ELN risk stratification of molecular, genetic and cytogenetic alterations.

Risk Group	Subsets
Favorable	t(8;21)(q22;q22); *RUNX1-RUNX1T1*
	inv(16)(p13.1q22) or t(16;16)(p13.1;q22); *CBFB-MYH11*
	Mutated *NPM1* without *FLT3*-ITD (normal karyotype)
	Biallelic mutated *CEBPA* (normal karyotype)
Intermediate-I	Mutated *NPM1* and *FLT3*-ITD (normal karyotype)
	Wild-type *NPM1* and *FLT3*-ITD (normal karyotype)
	Wild-type *NPM1* without *FLT3*-ITD (normal karyotype)
Intermediate-II	t(9;11)(p22;q23); *MLLT3-KMT2A*
	Cytogenetic abnormalities not classified as favorable
	or adverse
Adverse	inv(3)(q21q26.2) or t(3;3)(q21;q26.2); *GATA2-MECO (EVI1)*
	t(6;9)(p23;q34); *DEK-NUP214*
	t(v;11)(v;q23); *KMT2A* rearranged
	–5 or del(5q); –7; abnl(17p); complex karyotype *

Abbreviations: ITD, internal tandem duplication; * A complex karyotype is defined as three or more chromosome abnormalities; in the absence of one of the WHO designated recurring translocations; or inversions: t(8;21), inv(16) or t(16;16), t(15;17), t(9;11), t(v;11)(v;q23), t(6;9), inv(3) or t(3;3).
